# Long-Term Care Financing for an Aging Population: The Experience of Singapore

**DOI:** 10.1093/geroni/igaa057.101

**Published:** 2020-12-16

**Authors:** Ngee Choon Chia, Huijun Cynthia Chen

**Affiliations:** National University of Singapore, Singapore, Singapore

## Abstract

Singapore has a rapidly aging population. Long-term care (LTC) is one of the largest financial risks facing elderly in Singapore. Singapore implemented Eldershield, a long-term care insurance scheme which provided defined cash benefit payouts in the event of severe disability; but capped at a maximum of six years. Eldershield enrolled people at age 40, but offered an opt-out option. As of 2015, 65% of those aged 40 to 83 opted to be covered by Eldershield, making Singapore as having the highest voluntary LTC insurance rate in the world. This paper uses an actuarial multi-state disability model and calibrates the transition probabilities and duration-of-stay at various health (disability) states to assess the adequacy and comprehensiveness of Eldershield. The time-limited cash benefit design in Eldershield helped defray about 13% of LTC costs. Removing the time cap will help defray 23% and 26% of the LTC costs for elderly male and female respectively. Furthermore, the simulation results demonstrate that relaxing the trigger benefit and having staggered payouts will improve the adequacy of long-term care insurance. The experience of Singapore’s LTC insurance offers insights into the challenges of designing an insurance that tends to occur at higher age and insuring against a cost that could range from zero to a significantly large sum over a long period. Even with the enhanced Careshield Life, which provides cash payouts for life, other policy designs, for example caregiver grants, may be needed to ensure more adequate financing of long-term care.

